# Universal law for the vibrational density of states of liquids

**DOI:** 10.1073/pnas.2022303118

**Published:** 2021-01-25

**Authors:** Alessio Zaccone, Matteo Baggioli

**Affiliations:** ^a^Department of Physics “A. Pontremoli,” University of Milan, 20133 Milan, Italy;; ^b^Department of Chemical Engineering and Biotechnology, University of Cambridge, CB3 0AS Cambridge, United Kingdom;; ^c^Cavendish Laboratory, University of Cambridge, CB3 0HE Cambridge, United Kingdom;; ^d^Instituto de Fisica Teorica, Consejo Superior de Investigaciones Científicas, Universidad Autonoma de Madrid, 28049 Madrid, Spain;; ^e^Wilczek Quantum Center, School of Physics and Astronomy, Shanghai Jiao Tong University, Shanghai 200240, China;; ^f^Shanghai Research Center for Quantum Sciences, Shanghai 201315, China

**Keywords:** liquids, vibrational properties, unstable states, instantaneous normal modes

## Abstract

An analytical derivation of the vibrational density of states (DOS) of liquids, and, in particular, of its characteristic linear in frequency low-energy regime, has always been elusive because of the presence of an infinite set of purely imaginary modes—the instantaneous normal modes (INMs). By combining an analytic continuation of the Plemelj identity to the complex plane with the overdamped dynamics of the INMs, we derive a closed-form analytic expression for the low-frequency DOS of liquids. The obtained result explains, from first principles, the widely observed linear in frequency term of the DOS in liquids, whose slope appears to increase with the average lifetime of the INMs. The analytic results are robustly confirmed by fitting simulations data for Lennard-Jones liquids, and they also recover the Arrhenius law for the average relaxation time of the INMs, as expected.

In quantum physics, an exponentially decaying state is characterized by a complex value of the frequency, with the imaginary part giving the lifetime of the particle and its decay probability (1). Well-known examples of unstable states with overwhelming imaginary part arise in the nuclear α-decay (Gamow states) and, in particle physics—the W and $Z^{0}$ bosons (2)—where they are usually called “resonances.” Moreover, within hydrodynamics and gravitational theories, these excitations are labeled “quasinormal modes” (3), and they are the responsible for the black holes’ ringdown recently observed by LIGO Scientific Collaboration and Virgo Collaboration (4). In condensed matter physics, states with imaginary frequency arise frequently in disordered dissipative systems, in the form of purely relaxational overdamped modes, and even in heated crystals (5). They play a crucial role in liquids and glasses, where they are often called instantaneous normal modes (INMs) (6–8) and correspond to saddle points, with negative eigenvalues, in the energy landscape (9–11).

In all cases, defining the spectrum, or density of states (DOS), of systems that contain saddle points is challenging (12). The major obstacle resides in the fact that the Plemelj identity, that formally provides the connection between the propagator and the DOS, is defined on the real axis; hence only states with real frequencies ω, and positive eigenvalues $\lambda =\omega^{2}$ of the Hessian, are allowed. As a consequence, so far, it has not been possible to analytically derive the DOS of systems with imaginary modes from a physical model, and the DOS of systems with INMs has been computed analytically only in one dimension where negative eigenvalues are absent (13).

In liquids [broadly defined to include plasma (14)], due to the presence of a large quantity of overdamped, exponentially decaying modes with purely imaginary frequency (the INMs), this problem has hampered the derivation of a universal law for the DOS of vibrational excitations, g(ω). This is in stark contrast with the case of low-temperature crystals, where the vibrational modes are all real, and the Debye law $g\left(\omega \right)\approx \omega^{2}$ has served as a fundamental law for the DOS of solids since 1912. In solid glasses, the Debye law is still valid at the lowest frequencies, although hybridized with $\omega^{4}$ modes due to anharmonicity (15, 16). In liquids, we know from numerical simulations (7) that, as highlighted in ref. 17, g(ω)≈ω at low frequency, but none of the theoretical models have been able to reproduce this law analytically. This is due to the impossibility of including modes with negative eigenvalues which dominate the low-energy part of the spectrum.

Here we provide a solution to this long-standing problem, by developing the fundamental law for the DOS of liquids, which takes the imaginary modes into account analytically. The key step in our derivation is the analytic continuation of the Plemelj identity to the complex plane. This leads to the possibility of defining a DOS for systems with imaginary frequency/energy. Our analytical model is successfully tested against numerical simulations from the literature on model (Lennard-Jones [LJ]) liquids.

The vibrational DOS of condensed matter systems is defined as

$$g\left(\omega \right)=\frac{1}{3\;N}\underset{j}{\sum }\delta \left(\omega -\omega_{j}\right),$$ [1]

with the index j labeling different normal modes. Here N denotes the total number of atoms in the medium. We consider decaying modes which are purely relaxational, as they arise, for example, from an overdamped Langevin dynamics in a liquid,

$$\frac{dv}{dt}=-\Gamma \;v,\;\Gamma =1/\tau ,$$ [2]

where v is the particle velocity, and τ is the relaxation time. The Langevin differential operator, associated with Eq. **2**, is given by L ≔ d/dt+Γ, and the corresponding Green’s function is obtained by solving L G(t,x)=δ(t,x). After Fourier transforming the previous identity, we obtain the following form for the Green’s function:

$$G\left(\omega \right)=\frac{1}{i\;\omega +\Gamma }.$$ [3]

In order to relate the Green function for the single INM Eq. **3** with the total DOS Eq. **1**, we need to use the generalized form of Plemelj identity (see refs. 18 and 19) valid for integration paths that do not necessarily lie on the real line,

$$\frac{1}{z-z^{\prime }+i\;0^{+}}=P\left(\frac{1}{z-z^{\prime }}\right)-i\;\pi \;\delta \left(z-z^{\prime }\right),$$ [4]

where z and $z^{\prime }$ are arbitrary points in the complex plane region $D^{+}$, which coincides with the complex plane C minus the wedge −π/4<arg(z)<5π/4, and P indicates the Cauchy principal value. This limitation arises upon considering the derivation of the Plemelj identity via distribution theory. The starting point of the derivation is usually given by an integral,

$$\int_{0}^{\infty }dx\;e^{-izx}e^{-\epsilon x},$$

where, clearly, as long as z is real, no fundamental problem appears, and the integral converges everywhere. Typically (20), one then proceeds by showing that this integral is equal to what one obtains upon applying the Fourier transform to the Heaviside function in S (the space of Schwartz distributions) (21), which then leads to Eq. **4** above, with $z,z^{\prime }$ as all real variables.

Now, the analytic continuation of this integral, that is, promoting z to be a complex variable, leads to a divergence for Im z<0. Following Zeldovich (1), the Gaussian regularization

$$J\left(z,\lambda \right)=\int_{0}^{\infty }dx\;e^{-\lambda x^{2}}e^{izx}$$ [5]

can be used (upon subsequently taking the limit $\lambda \to 0^{+}$). Following ref. 18, one can show that Eq. **5** converges everywhere in the complex plane apart from the wedge defined by −π/4<arg(z)<5π/4. Hence, one retrieves the generalized Plemelj identity Eq. **4** valid for arbitrary pathways in the allowed complex plane region $D^{+}$.

We therefore write

$$G\left(z\right)=\frac{1}{\left(\xi +i\;\omega \right)-\left(-\Gamma +\xi \right)+i\;0^{+}},$$ [6]

thus identifying z≡i ω+ξ, and $z^{\prime }\equiv -\Gamma +\xi$, where ξ is real. Next, we apply the generalized Plemelj identity (Eq. **4**) to G(z) to evaluate the DOS,

$$\frac{1}{\left(\xi +i\;\omega \right)-\left(-\Gamma +\xi \right)+i\;0^{+}}=P\left(\frac{1}{z-z^{\prime }}\right)-i\;\pi \;\delta \left(z-z^{\prime }\right),$$ [7]

and, upon taking the imaginary part of the left-hand side, we thus obtain

$$g\left(z\right)\equiv \delta \left(z-z^{\prime }\right)=-\frac{1}{3\;\pi \;N}\;\text{Im}\left[\frac{1}{i\omega -\left(-\Gamma \right)+i\;0^{+}}\right]$$ [8]

$$=\frac{1}{3\;\pi \;N}\;\frac{\omega }{\omega^{2}+\Gamma^{2}}.$$ [9]

The above Eq. **9** is our main result and provides a simple yet universal law for the DOS of liquids. Eq. **9** is the density of states for a single INM with Green function Eq. **3**. Given the linearity of the problem, we can generalize the result to a set of j INMs with different relaxation times as

$$g_{\text{total}}=\frac{1}{3\;\pi \;N}\;\underset{j}{\sum }\;\frac{\omega }{\omega^{2}+\Gamma_{j}^{2}},$$ [10]

where $\Gamma_{j}$ is the relaxation rate of the jth INM.

Expanding Eq. **10** in the limit of low frequency, $\omega \ll \Gamma_{j}$, we obtain

$$g\left(\omega \right)=\alpha \;\omega +O\left(\omega^{3}\right),\;\alpha =\underset{j}{\sum }\;\frac{1}{3\;\pi \;N\;\Gamma_{j}^{2}},$$ [11]

which recovers a common trend observed in many molecular simulation studies of liquids, where α is treated as a fitting parameter (17, 22).

In Eq. **11**, the various relaxation rates sum up in parallel,

$$\frac{1}{\Gamma_{\text{total}}^{2}}=\underset{j}{\sum }\;\frac{1}{\Gamma_{j}^{2}},$$ [12]

implying that, in the presence of a separation of scales $\Gamma^{*}\ll \Gamma_{2},\Gamma_{3},\ldots \;$, the average relaxation rate Γ would be given by the smallest of them, that is, $\Gamma^{*}$. This is tantamount to saying that the low-frequency dynamics is governed by the longest living imaginary mode—a well-known result in the realm of hydrodynamics and effective field theory around equilibrium. In the rest of the paper, whenever we will discuss the relaxation rate, we will mean the total one defined in Eq. **12**.

We now present predictions of the main result of our paper, Eq. **11**, in comparison with numerical simulations data of simple liquids, that is, the LJ system. It is worth recalling that g(ω) is obtained numerically (from diagonalization of the Hessian matrix of instantaneous snapshots of particle positions) by retaining also the imaginary frequencies, because g(ω)≡g(|ω|) (6). Hence, in the following, ω stands for the absolute value of the excitation frequency.

In Fig. 1, we show the comparison between the model predictions and original MD simulations data of LJ liquids from ref. 17. The model Eq. **11** uses an effective relaxation time Γ as a fitting parameter, besides the normalization prefactor.

**Fig. 1.**
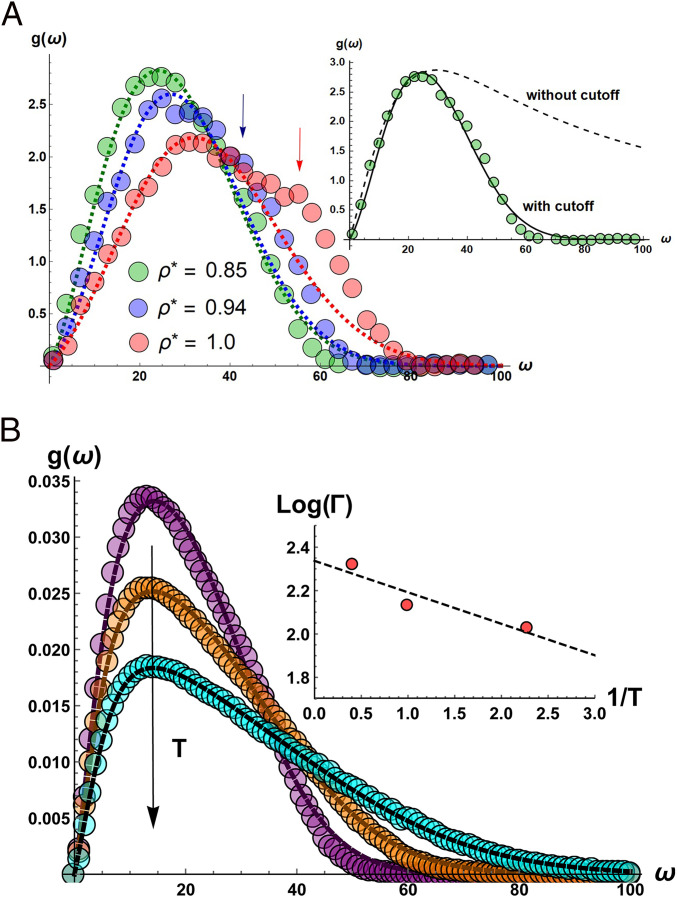
(*A*) Data of the DOS taken from ref. 17 and fitted with the analytic function Eq. **9**. A Gaussian (Debye) cutoff is added to take into account the large frequency fall-off. The arrows indicate the presence of possible relics of the van Hove singularities (which are not captured by our model) upon moving toward the solid phase. The original simulations were done using the standard LJ potential calibrated for Argon, and with N in the range 108 to 256. See ref. 17 for more details of the simulation protocol. *Inset* shows the fit without the Gaussian cutoff. (*B*) Data of the DOS of binary Kob–Andersen LJ liquids taken from ref. 23 and fitted with the analytical formula Eq. **9**. A Gaussian (Debye) cutoff is added to take into account the large frequency fall-off. *Inset* shows that the fitted relaxation time Γ in Eq. **11** as a function of temperature T behaves according to the Arrhenius law, as expected for equilibrium liquids (17). The size in the original simulations data was set to *N* = 1,000, and the DOS was averaged over 100 independent realizations. Simulations were performed with a standard Nosé–Hoover thermostat in the NVT ensemble.

The simulation data can be nicely fitted using Eq. **11** with just two parameters up to the maximum of the DOS, after which a Gaussian cutoff $∼e^{-{\left(\omega /\omega_{D}\right)}^{2}}$ is needed to capture the fall-off [note that, in low-T solid glasses, a simple exponential cutoff is instead used (24)]. In the dataset at the highest density, a small peak becomes visible, which is a relic of a pseudo-van Hove singularity upon approaching the solid state.

In Fig. 1, we compare predictions of Eq. **11** with the more recent simulations data from ref. 23, also for the LJ liquid. Also in this case, excellent agreement between Eq. **11** and simulations is found. As a further confirmation of the validity of our Eq. **11**, we have also checked that the mean relaxation time Γ follows the Arrhenius law as a function of T, as expected for equilibrium liquids (17), as shown in Fig. 1 *A*, *Inset*. We found that the activation energy for these relaxations is ∼0.15ϵ, where ϵ is the depth of the LJ attractive well, whereas the attempt frequency is ∼10ϵ/ℏ, consistently about 10 times the escape rate from the LJ well. Since Γ corresponds to the frequency of the maximum of the DOS (obtainable by setting the derivative of the summand in Eq. **10** equal to zero), this estimate gives an upper bound for the thermally activated relaxation processes from anharmonic saddle points in the energy landscape.

This comparison shows that, especially in the low-frequency region of the DOS up to the maximum, the spectrum is dominated by the relaxational modes or INMs. At higher frequencies, also, phonons are present in the DOS (25), which are not described by our minimal model but can be included in future work. At present, the mean relaxation time Γ acts as an effective parameter, which effectively takes into account, also, other vibrational excitations that are not explicitly implemented in the model and may become important around the frequency of the maximum and above.

In sum, the proposed theoretical model provides a universal law g(ω)∼ω for the low-frequency DOS of liquids, in agreement with observations from many simulations studies in the literature (6, 7, 17, 23). This law plays, for liquids, the same pivotal role that the Debye law $g\left(\omega \right)∼\omega^{2}$ plays for solids, and it explains, for example, that the liquids are mechanically unstable [by leading to diverging negative nonaffine contributions to the shear modulus (26)] and that INMs may play a role, also, for the thermal properties of solids. Furthermore, it could explain the observation of DOS scalings $∼\omega^{\alpha }$ with α∈[1,2] in glasses (27) and other complex systems. This law has not been derived before, due to the difficulty of dealing with the imaginary frequencies [associated with saddle points in the energy landscape (9, 10)] that dominate the low-frequency spectrum of liquids. In this work, we have solved this problem by analytically continuing the Plemelj identity to the complex plane using input from recent developments (18, 19). This methodology is general and extends far beyond the case of liquids or glasses. Further applications of our result will lead to the possibility of analytically describing the DOS of unstable states in quantum mechanics and the spectrum of black holes where imaginary modes and quasinormal modes play an important role.

## References

[1] Y. B. Zeldovich, On the theory of unstable states. Sov. Phys. JETP 12, 542 (1961).

[2] R. G. Stuart, Unstable particles. arXiv [Preprint] (1995). https://arxiv.org/abs/hep–ph/9504308 (Accessed 19 January 2021).

[3] E. Berti, V. Cardoso, A. O. Starinets, Quasinormal modes of black holes and black branes. Class. Quant. Grav. 26, 163001 (2009).

[4] R. Abbott, Gw190521: A binary black hole merger with a total mass of $150\;M_{⊙}$. Phys. Rev. Lett. 125, 101102 (2020). 32955328 10.1103/PhysRevLett.125.101102

[5] B. Madan, T. Keyes, Unstable modes in liquids density of states, potential energy, and heat capacity. J. Chem. Phys. 98, 3342–3350 (1993).

[6] T. Keyes, Instantaneous normal mode approach to liquid state dynamics. J. Phys. Chem. 101, 2921–2930 (1997).

[7] R. M. Stratt, The instantaneous normal modes of liquids. Acc. Chem. Res. 28, 201–207 (1995).

[8] G. V. Vijayadamodar, A. Nitzan, On the application of instantaneous normal mode analysis to long time dynamics of liquids. J. Chem. Phys. 103, 2169–2177 (1995).

[9] K. Broderix, K. K. Bhattacharya, A. Cavagna, A. Zippelius, I. Giardina, Energy landscape of a Lennard-Jones liquid: Statistics of stationary points. Phys. Rev. Lett. 85, 5360–5363 (2000). 11135996 10.1103/PhysRevLett.85.5360

[10] M. S. Shell, P. G. Debenedetti, A. Z. Panagiotopoulos, Saddles in the energy landscape: Extensivity and thermodynamic formalism. Phys. Rev. Lett. 92, 035506 (2004). 14753888 10.1103/PhysRevLett.92.035506

[11] E. La Nave, A. Scala, F. W. Starr, F. Sciortino, H. E. Stanley, Instantaneous normal mode analysis of supercooled water. Phys. Rev. Lett. 84, 4605–4608 (2000). 10990751 10.1103/PhysRevLett.84.4605

[12] T. S. Grigera, V. Martín-Mayor, G. Parisi, P. Verrocchio, Phonon interpretation of the ‘boson peak’ in supercooled liquids. Nature 422, 289–292 (2003). 12646916 10.1038/nature01475

[13] A. Cavagna, I. Giardina, G. Parisi, Analytic computation of the instantaneous normal modes spectrum in low-density liquids. Phys. Rev. Lett. 83, 108–111 (1999).

[14] M. S. Murillo, Ultrafast dynamics of neutral, ultracold plasmas. Phys. Plasmas 14, 055702 (2007).

[15] V. L. Gurevich, D. A. Parshin, H. R. Schober, Anharmonicity, vibrational instability, and the boson peak in glasses. Phys. Rev. B 67, 094203 (2003).

[16] E. Lerner, G. Düring, E. Bouchbinder, Statistics and properties of low-frequency vibrational modes in structural glasses. Phys. Rev. Lett. 117, 035501 (2016). 27472122 10.1103/PhysRevLett.117.035501

[17] E. Rabani, J. D. Gezelter, B. J. Berne, Calculating the hopping rate for self-diffusion on rough potential energy surfaces: Cage correlations. J. Chem. Phys. 107, 6867–6876 (1997).

[18] J. Julve, R. Cepedello, F. J. de Urries, The complex Dirac Delta, Plemelj formula, and integral representations. arXiv [Preprint] (2016). https://arxiv.org/abs/1603.05530 (Accessed 19 January 2021).

[19] J. Julve, F. J. de Urries, Inner products of resonance solutions in 1d quantum barriers. J. Phys. Math. Theor. 43, 175301 (2010).

[20] V. Vladimirov, Distributions en Physique Mathematique (Editions de Moscou, 1979).

[21] L. Schwartz, Theorie des Distributions (Hermann, Paris, France, 1966).

[22] J. Daligault, Universal character of atomic motions at the liquid-solid transition. arXiv [Preprint] (2020). http://arxiv.org/abs/2009.14718 (Accessed 19 January 2021).

[23] W. Zhang, J. F. Douglas, F. W. Starr, What does the instantaneous normal mode spectrum tell us about dynamical heterogeneity in glass-forming fluids? J. Chem. Phys. 151, 184904 (2019). 31731864 10.1063/1.5127821

[24] A. I. Chumakov, Collective nature of the boson peak and universal transboson dynamics of glasses. Phys. Rev. Lett. 92, 245508 (2004). 15245100 10.1103/PhysRevLett.92.245508

[25] Y. D. Fomin, Dynamics, thermodynamics and structure of liquids and supercritical fluids: Crossover at the Frenkel line. J. Phys. Condens. Matter 30, 134003 (2018). 29443011 10.1088/1361-648X/aaaf39

[26] V. V. Palyulin, Parameter-free predictions of the viscoelastic response of glassy polymers from non-affine lattice dynamics. Soft Matter 14, 8475–8482 (2018). 30152833 10.1039/c8sm01468j

[27] J. B. Suck, Dynamical structure factor and frequency distribution of the metallic glass Cu46Zr54 at room temperature. J. Phys. C Solid State Phys. 13, L167–L172 (1980).

